# Classical and Novel Biomarkers for Cardiovascular Risk Prediction in the United States

**DOI:** 10.2188/jea.JE20120157

**Published:** 2013-05-05

**Authors:** Aaron R. Folsom

**Affiliations:** Division of Epidemiology and Community Health, School of Public Health, University of Minnesota, Minneapolis, MN, USA

**Keywords:** risk factors, coronary disease, cardiovascular disease, epidemiology

## Abstract

Cardiovascular risk prediction models based on classical risk factors identified in epidemiologic cohort studies are useful in primary prevention of cardiovascular disease in individuals. This article briefly reviews aspects of cardiovascular risk prediction in the United States and efforts to evaluate novel risk factors. Even though many novel risk markers have been found to be associated with cardiovascular disease, few appear to improve risk prediction beyond the powerful, classical risk factors. A recent US consensus panel concluded that clinical measurement of certain novel markers for risk prediction was reasonable, namely, hemoglobin A1c (in all adults), microalbuminuria (in patients with hypertension or diabetes), and C-reactive protein, lipoprotein-associated phospholipase, coronary calcium, carotid intima-media thickness, and ankle/brachial index (in patients deemed to be at intermediate cardiovascular risk, based on traditional risk factors).

## 1. CARDIOVASCULAR DISEASE RISK FACTORS AND PREVENTION

In the latter half of the twentieth century, epidemiologic studies identified many important causes of cardiovascular disease (CVD) operating at the population and individual levels. Discovery of these “classical” risk factors (high blood pressure, dyslipidemia, smoking, diabetes, physical inactivity, and Western diet), along with the development of effective population-wide and high-risk prevention approaches to risk factors,^1^ contributed to a substantial decline in CVD mortality in many developed countries. Interest in CVD prevention has expanded in the United States to the extent that the American Heart Association (AHA) now promotes not only primary prevention of CVD through control of classical risk factors but also “primordial prevention” (ie, avoidance of ever having risk factors) and “maintenance of low risk” (ie, maintaining optimal risk factor levels throughout life).^2^

## 2. PREDICTION OF CARDIOVASCULAR RISK IN INDIVIDUALS

Risk prediction equations derived from epidemiologic cohort studies have proved to be useful tools in primary prevention of CVD at the individual, clinical level.^3^^,^^4^ The Framingham equation for estimating 10-year risk of coronary heart disease (CHD) is the most widely used risk prediction model,^5^ although others exist.^6^^–^^9^ The Framingham model is based on the classical risk factors, namely, age, sex, blood pressure, low-density lipoprotein (LDL) and high-density lipoprotein (HDL) cholesterol, smoking, and sometimes diabetes. Clinical assessment of estimated 10-year CHD risk is promoted in order to guide hyperlipidemia treatment in the United States.^10^ Furthermore, the AHA recommends that for the purpose of CHD prevention clinicians should measure risk factors and calculate overall CHD risk in all adult patients.^11^ However, use of CHD risk prediction equations is far from universal in the United States, and physicians often simply count risk factors to characterize overall risk.

As reviewed elsewhere,^3^^,^^4^ some scientists have criticized the Framingham 10-year CHD risk estimation for (1) its focus on 10-year CHD risk rather than lifetime risk, (2) the strong contribution of age, which is not modifiable, to CHD prediction, (3) the uncertain generalizability of Framingham risk estimation to other populations, which seems to have been solved by population-specific recalibration,^12^ (4) a focus on CHD, rather than total CVD, which the Framingham investigators recently resolved with CVD risk equations,^13^ and (5) the suboptimal accuracy of risk prediction based on the limited set of classical risk factors. However, this concern that classical risk factors are insufficiently predictive is misguided and may have been perpetuated by a long-held belief that classical risk factors explained no more than 50% of CHD occurrence. Recent evidence based on population attributable risk calculations suggests that, in fact, 75% to 85% of CHD in the United States can be prevented by avoiding classical risk factors.^2^

## 3. WHICH NOVEL RISK FACTORS MIGHT IMPROVE CARDIOVASCULAR DISEASE PREDICTION?

Even though the classical risk factors for CVD are most important, cardiovascular epidemiologists have remained interested in identifying potential novel risk factors (Table 1). Identification of such factors could help clarify CVD pathophysiology, offer targets for intervention, or lead to improved risk stratification beyond that allowed by the Framingham equations. As Greenland pointed out,^14^ novel risk factors or biomarkers may be most useful for risk prediction and preventive decision making among patients at “intermediate” Framingham 10-year CHD risk. In contrast, novel risk factor measurement is clinically less useful in high- and low-risk patients. That is, patients at high risk of CHD (as determined using classical risk factors) require intervention regardless of the levels of novel biomarkers, and classically low-risk patients may need no intervention, even if novel biomarkers are elevated.

**Table 1.** Examples of novel biomarkers of potential interest in cardiovascular disease risk prediction

***Novel blood and urine markers***	
Lipid-related markers	Apolipoprotein A1
	Apolipoprotein B100
	Lipoprotein-associated phospholipase A2 (LpPLA_2_)
	Lipoprotein(a)

Renal function markers	Creatinine
	Cystatin-C

Metabolic markers	Adiponectin
	Leptin
	Insulin
	Glycosylated hemoglobin (HbA_1_C)

Coagulation markers	Fibrinogen
	D-dimer

Markers of vascular function and neurohumoral activity	(N-terminal pro) B-type natriuretic peptide
	Mid-regional pro-adrenomedullin
	Microalbuminuria

Inflammatory markers	C-reactive protein (CRP)
	Interleukin-6 (IL-6)

Markers of oxidative stress and antioxidants	Homocysteine
	Myeloperoxidase

Necrosis markers	Troponin I or T

***Atherosclerosis markers***	
Structural	Carotid intima-media thickness (IMT) and plaque measured by ultrasound
	Aortic and carotid plaque detected by MRI
	Coronary calcium (CAC) score measured by CT
	Ankle brachial index
	Pulse wave velocity
	Brachial vasoreactivity measured by ultrasound

Functional	Vascular compliance measured by radial tonometry
	Microvascular reactivity measured by fingertip tonometry

***Genetic markers***	Candidate or discovered single-nucleotide polymorphisms (SNPs)

Methods of determining whether a new CVD biomarker adds to risk prediction in epidemiologic cohort studies have received much attention recently. It is not enough to show a novel causal or noncausal biomarker is “independently associated” with CHD. A novel biomarker must add incrementally to CVD prediction equations beyond the classical risk factors, in terms of model performance, discrimination, and calibration and event reclassification^15^^–^^19^ (Table 2). Thus, the addition of a novel risk factor to an existing CVD risk prediction model should improve the C statistic, and the net reclassification index (NRI) should be sizable. (The C statistic is the area under the receiver operating characteristic [ROC] curve and is a measure that discriminates between those who developed disease and those who did not, based on ranks. The NRI for adding a novel risk factor to a prediction model is the net increase versus decrease in risk factor categories among those who developed disease, minus that among those who did not develop disease.) A novel biomarker must have a high risk ratio to contribute incrementally to the very good CHD prediction afforded by classical risk factors.^20^ Thus, to date, few novel biomarkers of CHD risk have been widely adopted for clinical use in the United States.

**Table 2.** Some measures of performance for prediction models

Aspect	Measure	Visualization
Overall performance	*R*^2^, Brier	Validation graph
Discrimination	C statistic	Receiver operating characteristic (ROC) curve
Calibration	Calibration slope	Calibration or validation graph
	Hosmer-Lemeshow test	
Reclassification	Reclassification table	Cross-table or scatterplot
	Net reclassification index (NRI)	
	Integrated discrimination index (IDI)	Box plots for 2 models (1 with and 1 without a marker)

Derived from Reference ^22^.

Although this report is not a systematic review, Tables 3 to 6 show examples from recent cohort studies of the extent to which risk prediction models using classical risk factors, like the Framingham model, are improved by the addition of novel biomarkers. As reflected by a change in the C statistic of greater than 0.01 and an NRI of greater than 10%, structural and functional measures of subclinical atherosclerosis, like coronary artery calcium, tend to significantly improve prediction of CHD/CVD risk beyond classical risk factors (Table 3). As shown in Table 4, in most studies, inflammatory and hemostatic blood biomarkers tended to add only modestly beyond classical risk factors (change in C statistic, <0.01; NRI, <5%). Two blood biomarkers more specifically related to cardiac dysfunction—high-sensitivity troponin T or I and B-type natriuretic peptide (Table 5)—seem to predict CHD somewhat better than inflammatory and hemostatic markers. In contrast, although numerous CVD-related genetic loci have recently been identified,^21^ genetic markers currently seem to add little to CHD risk prediction models (Table 6).

**Table 3.** Improvement in CVD/CHD prediction from addition of novel atherosclerosis markers to classical risk factor prediction models

Study	Outcome	Markers Added^a^	Δ C statistic^b^	NRI^c^
MESA^23^	CHD	CAC	0.76 → 0.81	0.25
MESA^24^	CVD	Small-artery elasticity	0.777 → 0.782	0.11
Heinz Nixdorf^25^	CHD	CAC	0.68 → 0.75	0.22
Rotterdam^26^	CHD	CAC	0.72 → 0.76	0.14
ARIC^27^	CHD	Carotid IMT or plaque	0.74 → 0.76	0.10
ABI Collaboration^42^	CHD	ABI (men)	0.646 → 0.655	
		ABI (women)	0.605 → 0.658	

^a^CVD, cardiovascular disease; CHD, coronary heart disease; CAC, coronary artery calcium; IMT, intima-media thickness; ABI, ankle brachial index.

^b^Change in C statistic from addition of the novel marker to a classical risk factor model.

^c^Overall net reclassification index (NRI), based on 3 categories^23^^,^^25^^,^^26^ or 4 categories^24^^,^^27^ of risk.

**Table 6.** Improvement in CVD/CHD prediction from addition of SNPs to classical risk factor prediction models

Study	Outcome	Markers Added^a^	Δ C Statistic^b^	NRI^c^
ARIC^39^	CHD	9p21 SNP	0.782 → 0.786	0.008
Scandinavia^40^	CHD	13 SNP Score	0.87 → 0.87	0.02
Women’s Genome Health^41^	CVD	101 SNPs (with FHx in model)	0.796 → 0.796	0.005
Malmö^32^	CVD	9 lipid SNPs	0.80 → 0.80	

^a^CVD, cardiovascular disease; CHD, coronary heart disease; SNP, single-nucleotide polymorphism; FHx, family history.

^b^Change in C statistic from addition of the novel marker to a classical risk factor model.

^c^Overall net reclassification index (NRI), based on 4 categories^39^^–^^41^ of risk.

**Table 4.** Improvement in CVD/CHD prediction from addition of novel inflammatory or hemostatic markers to classical risk factor prediction models

Study	Outcome	Markers Added^a^	Δ C statistic^b^	NRI^c^
Physicians Health^28^	CVD	CRP, FHx	0.699 → 0.708	0.05
ARIC^29^^,^^30^	CHD	CRP	0.767 → 0.770	
		IL-6	0.773 → 0.783	
		D-dimer	0.805 → 0.803	
		Fibrinogen (WM)	0.688 → 0.699	
		Fibrinogen (WW)	0.793 → 0.795	
Framingham^31^	CVD	CRP	0.795 → 0.799	0.06
	CHD	CRP	0.863 → 0.865	0.12
Malmö^35^	CVD	CRP, NT-pro BNP	0.758 → 0.765	0.00
Women’s Health Initiative^33^	CHD	IL-6, D-dimer, FVIII, vWF, hcy	0.715 → 0.731	0.06

^a^CVD, cardiovascular disease; CHD, coronary heart disease; FHx, family history; IL-6, interleukin-6; CRP, C-reactive protein; WM, white men; WW, white women; BNP, B-type natriuretic peptide; FVIII, factor VIII; vWF, von Willebrand factor; hcy, homocysteine.

^b^Change in C statistic from addition of the novel marker to a classical risk factor model.

^c^Overall net reclassification index (NRI), based on 3 categories^31^^,^^33^^,^^35^ or 4 categories^28^ of risk.

**Table 5.** Improvement in CVD/CHD prediction from addition of novel cardiac markers to classical risk factor prediction models

Study	Outcome	Markers Added^a^	Δ C Statistic^b^	NRI^c^
Framingham^34^	CVD	BNP, albumin/creat	0.76 → 0.77	
Malmö^35^	CHD	MR-proADM, NT-pro BNP	0.760 → 0.769	0.05
ARIC^36^	CHD	hs-Troponin T	0.715 → 0.724	0.05
MORGAM^37^	CVD	NT-pro BNP, CRP, Troponin I	0.67 → 0.70	0.11
Uppsala men^38^	CVD death	Troponin I, NT-pro BNP, Cystatin C, CRP	0.69 → 0.75	0.26

^a^CVD, cardiovascular disease; CHD, coronary heart disease; BNP, B type natriuretic peptide; albumin/creat, urine albumin/creatinine; MR-proADM, mid-regional pro-adrenomedullin; CRP, C-reactive protein.

^b^Change in C statistic from addition of the novel marker to a classical risk factor model.

^c^Overall net reclassification index (NRI), based on 3 categories^35^^,^^38^ or 4 categories^36^^,^^37^ of risk.

## 4. RECENT US CONSENSUS OPINIONS ON MEASUREMENT OF NOVEL RISK MARKERS

In 2010, a joint task force of the American College of Cardiology and AHA^11^ issued guidance on which novel risk factors or biomarkers, in addition to classical risk factors, might be currently considered in CHD risk prediction (Table 7). The task force categorized family history as useful and hemoglobin A1c measurement as reasonable in all adults, and they categorized microalbuminuria assessment as reasonable in adults with hypertension or diabetes. With regard to more-novel biomarkers, the task force categorized measurement of C-reactive protein (CRP), lipoprotein-associated phospholipase A2 (LpPLA_2_), coronary calcium, carotid intima-media thickness, and ankle/brachial index to be reasonable for refining risk estimation and making clinical decisions in individuals initially classified as at intermediate CHD risk, using classical risk factors. They did not recommend assessing natriuretic peptides, apolipoproteins, or genetic markers, and they did not evaluate high-sensitivity troponin for its contribution to risk prediction. Additional evidence supporting the use of natriuretic peptides and troponin T or I in risk prediction appeared after the task force met.^36^^,^^37^

**Table 7.** ACC/AHA^a^ guideline on CVD risk assessment in asymptomatic adults

	Useful in All	Reasonable in All	Reasonable if CHD Risk is Intermediate	Not Recommended
Family Hx	✓			
HbA1c		✓		
Microalbuminuria		✓*	✓	
CRP			✓	
LpPLA_2_			✓	
Coronary Calcium			✓	
Carotid IMT			✓	
Ankle/Brachial Index			✓	
Brachial Vasoreactivity				✓
Natriuretic Peptides				✓
Apolipoproteins				✓
Genetic Testing				✓

^a^ACC, American College of Cardiology; AHA, American Heart Association; CVD, cardiovascular disease.

*In patients with hypertension or diabetes.

Source: Reference ^11^.

## 5. CONCLUSION

Although enthusiasm for research on novel biomarkers of CVD risk remains high in the United States, only a few such biomarkers have been accepted as clinically useful.
